# Larval zebrafish burn wound infection model reveals conserved innate immune responses against diverse pathogenic fungi

**DOI:** 10.1128/mbio.03480-24

**Published:** 2025-04-08

**Authors:** Nayanna M. Mercado Soto, Adam Horn, Nancy P. Keller, Anna Huttenlocher, Andrew S. Wagner

**Affiliations:** 1 Department of Medical Microbiology and Immunology, University of Wisconsin-Madison 5228, Madison, Wisconsin, USA; 2 Microbiology Doctoral Training Program (MDTP), University of Wisconsin-Madison 5228, Madison, Wisconsin, USA; 3 Department of Plant Pathology, University of Wisconsin-Madison 312673, Madison, Wisconsin, USA; 4 Department of Pediatrics, University of Wisconsin-Madison Department of Pediatrics 200763, Madison, Wisconsin, USA; 5 Department of Biological Sciences, Bowling Green State University 110004, Bowling Green, Ohio, USA; Universidade de Sao Paulo, Ribeirao Preto, Sao Paulo, Brazil

**Keywords:** fungi, *Aspergillus fumigatus*, *Candida albicans*, burn wound, zebrafish, innate immunity, β(1,3)-glucan

## Abstract

Secondary fungal infections represent a major complication following thermal injuries. However, the mechanisms of fungal colonization of burn tissue and how the host subsequently responds to fungi within this niche remain unclear. We have previously reported a zebrafish model of thermal injury that recapitulates many of the features of human burn wounds. Here, we characterize host-fungal interaction dynamics within the burn wound niche using two of the most common fungal pathogens isolated from burn injuries, *Aspergillus fumigatus* and *Candida albicans*. Both *A. fumigatus* and *C. albicans* colonize burned tissue in zebrafish larvae and induce a largely conserved innate immune response following colonization. Using drug-induced cell-depletion strategies and transgenic zebrafish lines with impaired innate immune function, we found that macrophages control fungal burden, whereas neutrophils primarily control invasive hyphal growth at the early stages of infection. However, we also found that loss of either immune cell can be compensated by the other at the later stages of infection and that fish with both macrophage and neutrophil deficiencies show more invasive hyphal growth that is sustained throughout the infection process, suggesting redundancy in their antifungal activities. Finally, we demonstrate that *C. albicans* strains with increased β(1,3)-glucan exposure are cleared faster from the burn wound, demonstrating a need for shielding this immunogenic cell wall epitope for the successful fungal colonization of burn tissue. Together, our findings support the use of zebrafish larvae as a model to study host-fungal interaction dynamics within burn wounds.

**IMPORTANCE:**

Secondary fungal infections within burn wound injuries are a significant problem that delays wound healing and increases the risk of patient mortality. Currently, little is known about how fungi colonize and infect burn tissue or how the host responds to pathogen presence. In this report, we expand upon an existing thermal injury model using zebrafish larvae to begin elucidating both the host immune response to fungal burn colonization and fungal mechanisms for persistence within burn tissue. We found that both *Aspergillus fumigatus* and *Candida albicans*, common fungal burn wound isolates, successfully colonize burn tissue and are effectively cleared in immunocompetent zebrafish by both macrophages and neutrophils. We also find that *C. albicans* mutants harboring mutations that impact their ability to evade host immune system recognition are cleared more readily from burn tissue. Collectively, our work highlights the efficacy of using zebrafish to study host-fungal interaction dynamics within burn wounds.

## INTRODUCTION

The World Health Organization (WHO) estimates that around 11 million burn injuries per year occur worldwide, with almost 200,000 resulting in patient death (1). Burn wound infections (BWIs) are the leading cause of mortality among burn wound patients and add an additional layer of complexity that can delay the healing process and further alter the immune response (2–4). BWIs are primarily caused by bacterial species like *Pseudomonas aeruginosa* and *Staphylococcus aureus* (2, 5) and are readily treated with antibiotics either following diagnosis or as a prophylactic (6, 7). However, the use of antibiotics can further disrupt the local microflora within the injury site and are thought to provide a platform for fungal infections to occur. BWIs caused by fungi are on the rise, and global reports have shown that up to 44% of severe burn wounds develop secondary infections from fungal species (4). Once colonized, the risk of developing systemic infections increases substantially, with mortality rates reported to be as high as 76% once dissemination occurs (8–10). Further complicating disease management, studies have shown that immune cells recruited to burn tissue can display defects in antimicrobial activity, such as reduced neutrophil oxidative burst and phagocytosis capacity, even though they are readily activated by pro-inflammatory cytokines and host damage-associated molecular patterns (11, 12). Despite the high prevalence and medical significance of fungal BWIs, little is known about how fungi successfully colonize burn tissue to cause disease or how the host subsequently responds to invading fungi at this niche. Indeed, most of the available data are based on clinical studies, highlighting the need for a robust infection model to further elucidate these dynamics.

Although fungal BWIs are understudied, *in vitro* and *in vivo* models do exist that have provided initial insights into the host response and therapeutic strategies following disease onset. For example, BWIs in human tissue explants have been recently established as an *ex vivo* model to study the host response to *Candida albicans* and have elegantly shown a role for neutrophils in responding to both burn damage and fungi (9). Animal burn models using mice, rats, pigs, and galleria have also been utilized to study drug efficacy following fungal burn wound colonization (13–15). However, these models have yet to be leveraged to extensively study the mechanisms of fungal colonization and host immune response, and many do not easily permit *in vivo* imaging of host-fungal interactions to better characterize disease dynamics. Zebrafish larvae, which are naturally transparent, provide an advantage for imaging infection kinetics *in vivo* (16–18). These vertebrates have a largely conserved innate immune response with humans, and zebrafish larvae have been established as a reliable model to study the inflammatory profile and tissue remodeling processes following burn injury (19, 20). In addition, we have shown that the inflammatory profile of burn wounds can be altered by *P. aeruginosa* colonization and have recently expanded upon the zebrafish burn model to show that thermal injuries can be colonized by the fungal pathogen *C. albicans* (21, 22). However, the mechanisms of fungal colonization and host-mediated clearance remain unknown.

Here, we characterize a fungal BWI model in zebrafish using two of the most common fungal pathogens isolated from burn wounds, *C. albicans* and the filamentous fungus *Aspergillus fumigatus* (5, 8, 10, 23, 24). Both *C. albicans* and *A. fumigatus* can colonize burn wounds as either yeast cells or spores, respectively, and undergo morphological transitions to hyphae to penetrate and invade neighboring tissues. We demonstrate that both species colonize the burn wound in zebrafish larvae and are successfully cleared in an immunocompetent host. We found that macrophages and neutrophils play synergistic and partially redundant roles in controlling fungal burden and hyphal formation and that infection in zebrafish with both deficient macrophages and neutrophils results in extensive colonization and invasive hyphal growth from the site of injury. We also show that fungal mutants with increased β(1,3)-glucan exposure are cleared faster from the burn site, indicating a need for shielding immunogenic epitopes in the fungal cell wall for persistent colonization to occur. Collectively, our data demonstrate conserved immune responses against two distinct fungal pathogens in the burn wound and highlight the potential of zebrafish larvae as a model to study fungal BWIs.

## RESULTS

### Pathogenic fungi colonize burn wounds in immunocompetent zebrafish larvae

**Fig 1:**
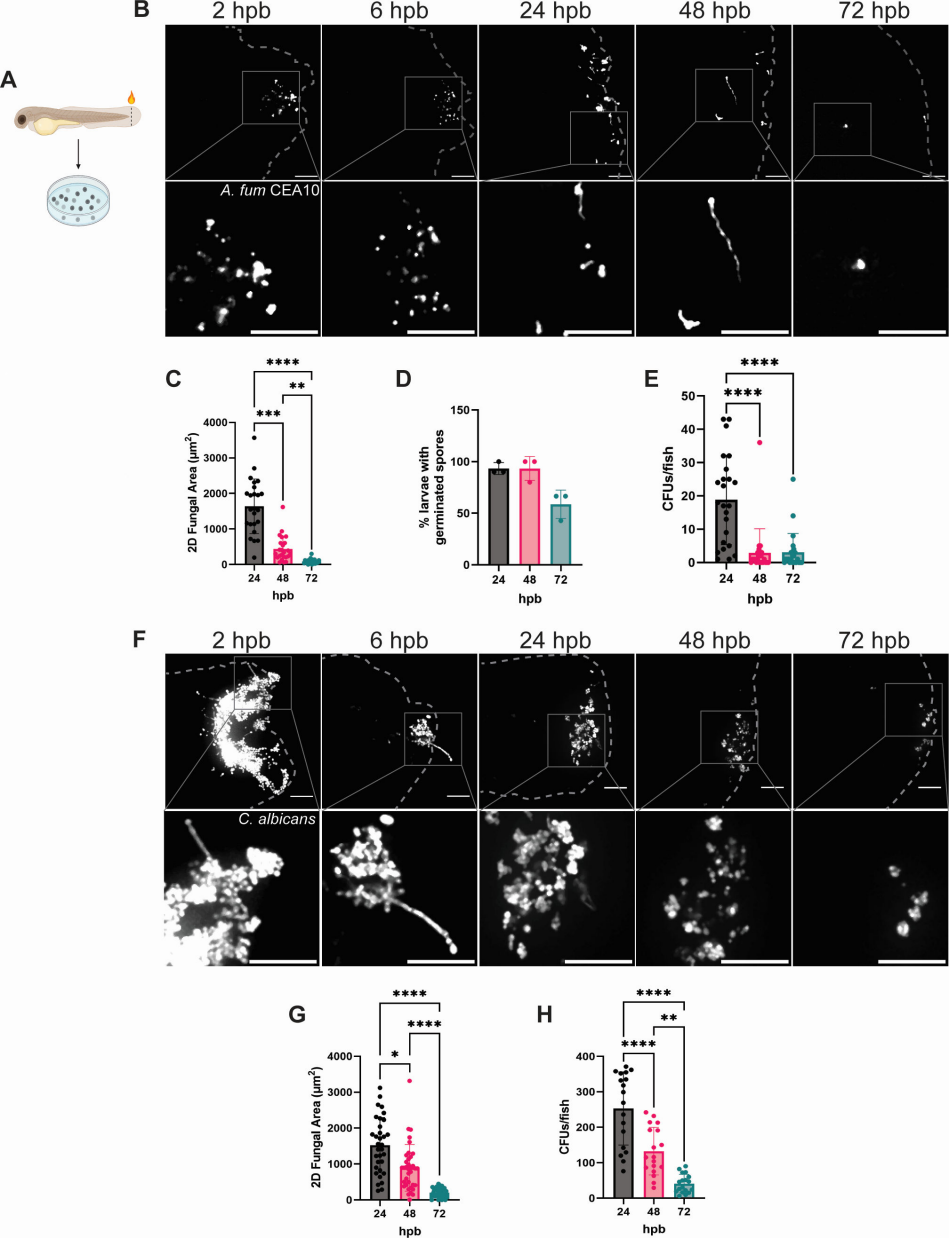
Immunocompetent fish clear fungi from the burn wound. (A) Fungal burn wound infection schematic. The tail fins of 3 days post-fertilization (dpf) larvae are injured using a cauterizer and immediately incubated in E3-MB media with 1 × 10^7^ fungal cells/mL. (B) Representative images of wild-type larvae infected with fluorescent (RFP) *A. fumigatus* CEA10 at 2, 6, 24, 48, and 72 h post-burn (hpb). Images represent maximum intensity projections of z-stacks, and the bottom panel represents zoomed versions of the original image (scale bar is 50 µm). Dashed gray lines outline tail fin edge, and the squares outline the portion of the image that is zoomed. (C) *A. fumigatus* 2D-fungal area from 24 to 72 hpb. (D) Percent of larvae with germinated *A. fumigatus* spores from 24 to 72 hpb. Each data point represents the percent of fish containing germinated spores from an individual experiment. (E) CFUs from *A. fumigatus* infected fish at 24–72 hpb. (F) Representative images of wild-type larvae infected with fluorescent (dTomato) *C. albicans* at 2, 6, 24, 48, and 72 h post-burn (hpb). Images represent maximum intensity projections of z-stacks, and the bottom panel represents zoomed versions of the original image (scale bar is 50 µm). Dashed gray lines outline tail fin edge, and the square outlines the portion of the image that was zoomed. (G) *C. albicans* 2D-fungal area and (H) CFUs/fish from 24 to 72 hpb. (For all graphs, each data point represents data from an individual fish, and results represent data pooled from three independent experiments. *n* = 24–30 larvae per condition from three independent experiments. *P < 0.05, **P < 0.01, ***P < 0.001, ****P < 0001, via either a one-way ANOVA with Tukey’s multiple comparisons test or a one-way ANOVA with a Kruskal-Wallis test.)

We have recently expanded on a preexisting burn wound injury model using 3 days post-fertilization (dpf) larvae to show that *C. albicans* can successfully colonize burn tissue in this model (22). However, characterization of the infection process and the subsequent host response has yet to be assessed. Furthermore, it is currently unknown if additional pathogenic fungi commonly isolated from BWIs are capable of infecting damaged tissue in a larval zebrafish burn model. To address this, we induced burn wound injury in 3 dpf zebrafish larvae via cauterization and incubated fish with either fluorescent-labeled spores of *Aspergillus fumigatus* or *Candida albicans* yeast to permit injury colonization (Fig. 1A). Both confocal microscopy images and plating for colony-forming units (CFUs) showed that *A. fumigatus* was able to successfully colonize the burn wound, with peak fungal burden at the 24 h time point, followed by a dramatic fungal clearance by 72 h post-burn (hpb) (Fig. 1B through E). Importantly, the infection was restricted to the burn wound area by 24 hpb, highlighting the need for tissue disruption for colonization to occur (Fig. S1A). In addition to colonization, germinated spores and/or hyphae were seen across all time points but were limited to small hyphal extensions localized to the tail fin (Fig. 1D). It should be noted that at time points prior to 24 hpb, there is an abundance of fungi superficially adhered to necrotic tissue of the burn wound, but by 24 hpb, fungal cells are interstitial within viable tissue. Therefore, we considered the 24 hpb time point as the start of stable colonization of the burn wound for further analysis.

The ability to colonize the burn wound was conserved by *C. albicans* (Fig. 1F). Strikingly, *C. albicans* seemed to persist longer, with the higher fungal burden compared with *A. fumigatus,* especially at 72 hpb (Fig. 1G and H). Similar to *A. fumigatus*, morphological transition to hyphae was seen during *C. albicans* infection, but the kinetics of these transitions differed. *A. fumigatus* germination was seen starting at 24 hpb, but *C. albicans* hyphae were observed as early as 2–6 hpb and were largely controlled by 24 hpb (Fig. 1F). This is consistent with previously published work in which *C. albicans* germination starts within 6 h post-infection (hpi) in a hindbrain ventricle zebrafish infection model, but by 24 hpi, the host immune system effectively controls hyphal growth (25).

### Fungal colonization increases immune cell recruitment to burns

**Fig 2:**
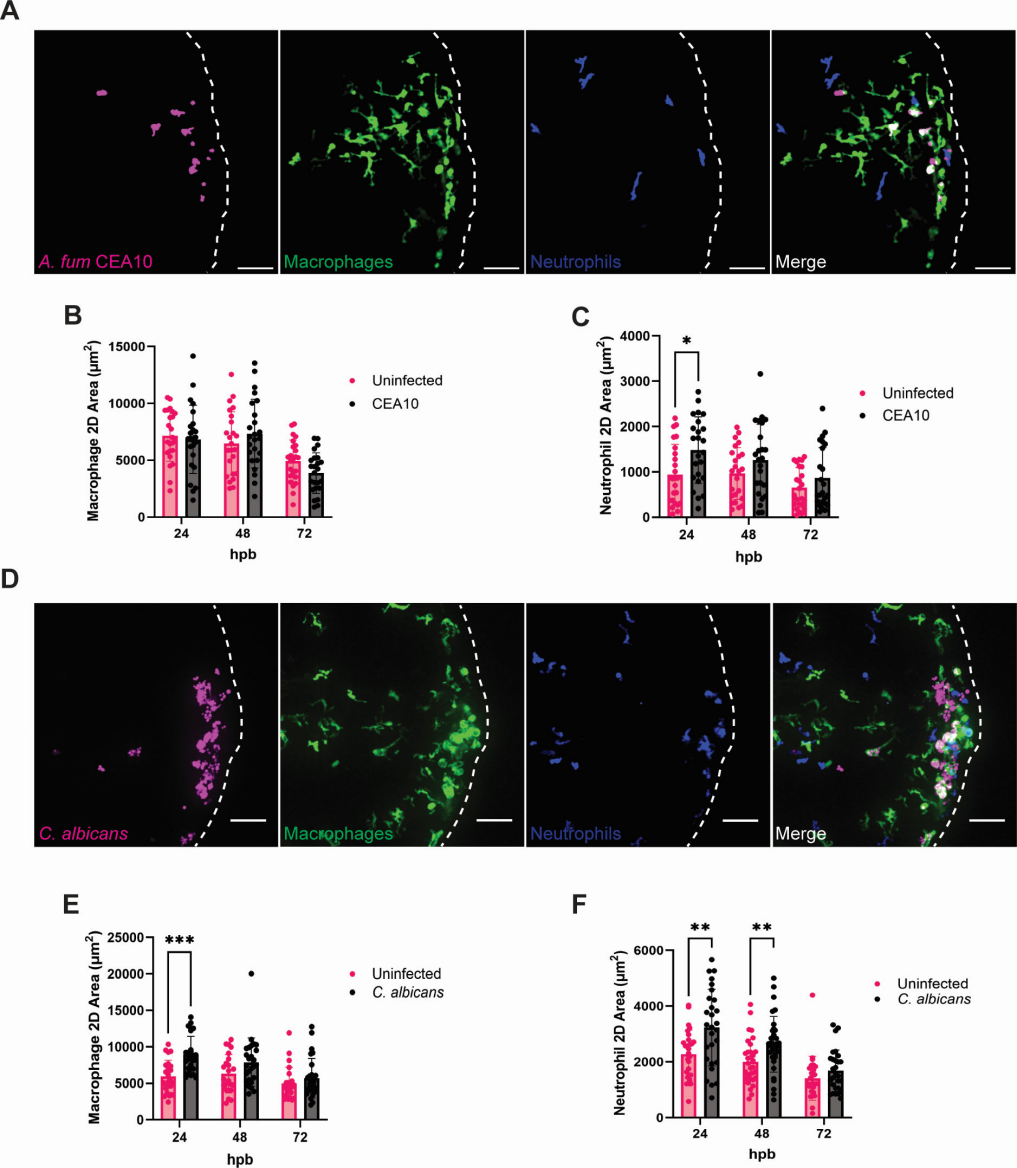
Fungal presence increases immune cell infiltration following thermal injury. (A-C) Wild-type larvae with fluorescent macrophages (*mpeg-GFP*) and neutrophils (*lyz-BFP*) were burned and infected with *A. fumigatus* CEA10 (RFP). Control larvae were injured but uninfected. Larvae were imaged at 24, 48, and 72 hpb. (A) Representative images of larvae infected with *A. fumigatus* CEA10 at 24 hpb. Images represent maximum intensity projections of z-stacks (scale bar is 50 µm). Dashed white lines outline tail fin edge. (B) 2D-macrophage area of *A. fumigatus* infected fish from 24 to 72 hpb. (C) 2D-neutrophil area of *A. fumigatus* infected fish from 24 to 72 hpb. (D–F) Wild-type larvae with fluorescent macrophages (*mpeg-GFP*) and neutrophils (*lyz-BFP*) were burned and infected with *C. albicans* (dTomato). (D) Representative images of larvae infected with *C. albicans* at 24 hpb. Images represent maximum intensity projections of z-stacks (scale bar is 50 µm). Dashed white lines outline tail fin edge. (E) 2D-macrophage area of *C. albicans* infected fish from 24 to 72 hpb. (F) 2D-neutrophil area of *C. albicans* infected fish from 24 to 72 hpb. (For all graphs, each data point represents data from an individual fish, and results represent data pooled from three independent experiments. *n* = 24–30 larvae per condition from three independent experiments. *P < 0.05, **P < 0.01, ***P < 0.001, via either multiple Student’s *t*-tests or multiple Mann-Whitney tests.)

In both humans and fish, macrophages and neutrophils are recruited to the burn wound and function to clear cellular debris from damaged and necrotic tissues (19, 21). To assess if fungal BWI further impacted immune cell recruitment, we burned and infected larvae with fluorescently labeled macrophages (*mpeg-GFP*) and neutrophils (*lyz-BFP*) and quantified leukocyte recruitment. By 24 hpb, macrophages and neutrophils are recruited to the burn wound during both *A. fumigatus* (Fig. 2A through C) and *C. albicans* (Fig. 2D through F) infection, and their numbers drop over a 72 h period in accordance with wound healing and fungal clearance. Like the inflammatory profile of the burn wound at basal levels (19), macrophages were the predominant cell type present during both fungal infections (Fig. 2B and E). Interestingly, at 24 hpb, fish infected with *C. albicans* showed increased levels of both macrophages and neutrophils at the tail fin when compared with uninfected burned fish (Fig. 2E and F), but only neutrophil levels were increased at 24 hpb during *A. fumigatus* infection (Fig. 2C). However, neither *C. albicans* nor *A. fumigatus* significantly impaired tail fin regeneration compared with the uninfected control following burn (Fig. S1B through D).

**Fig 3:**
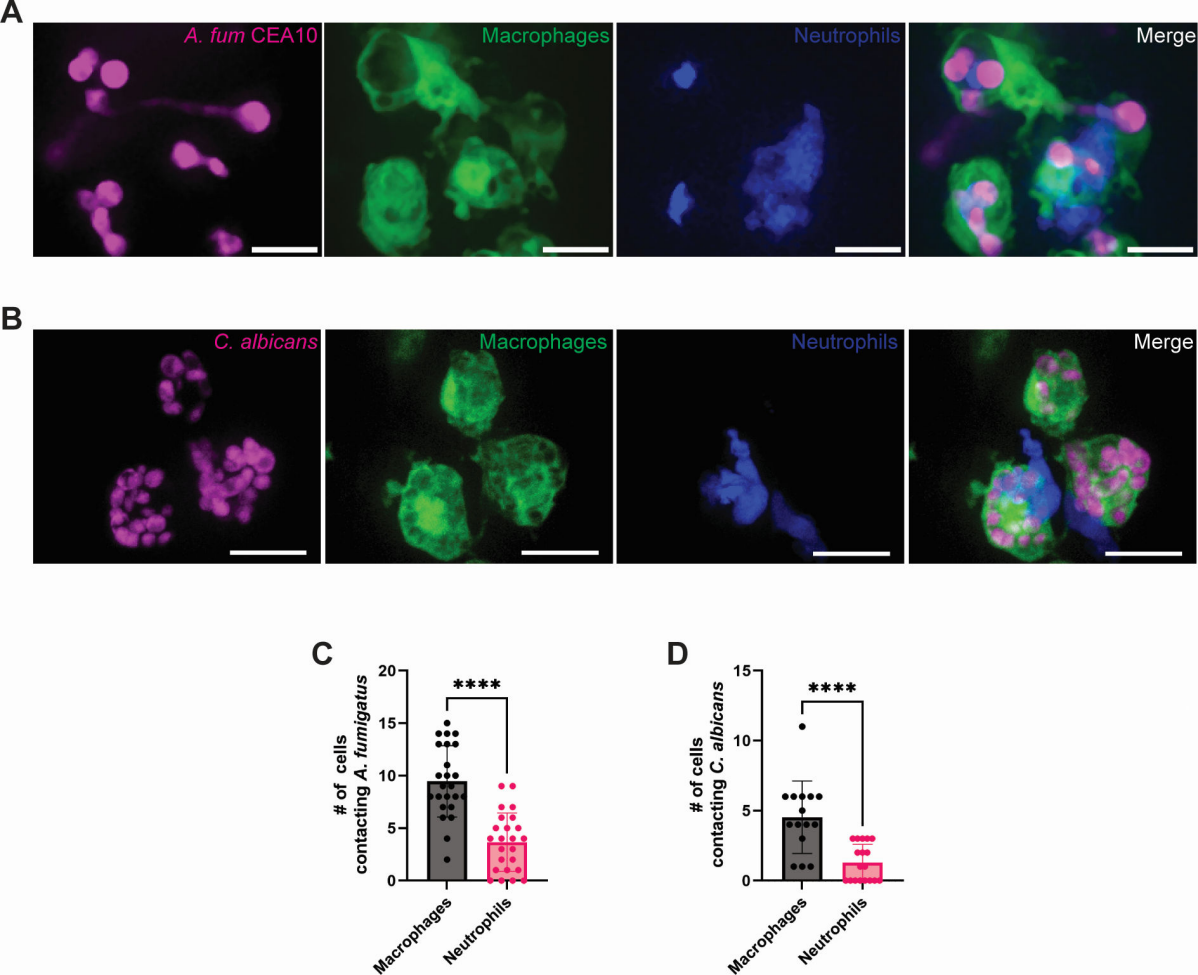
Macrophages primarily interact with fungi in burn tissue. (A-B) The 24 hpb representative images of wild-type larvae with fluorescent macrophages (*mpeg-GFP*) and neutrophils (*lyz-BFP*) inoculated with (A) *A. fumigatus* CEA10 spores (RFP) or (B) *C. albicans* yeast cells (dTomato). Images represent maximum intensity projections of z-stacks (scale bar is 10 µm for *A. fumigatus* and 15 µm for *C. albicans*) (C and D) Number of macrophages and neutrophils contacting (C) *A. fumigatus* cells and (D) *C. albicans* cells. (For all graphs, each data point represents data from an individual fish and results represent data pooled from three independent experiments. *n* = 24 larvae per condition from three independent experiments. *P* values were calculated by Student’s *t*-test for panel C and a Mann-Whitney test for panel D. ****P < 0001.)

To elucidate which phagocyte may be predominantly driving fungal clearance, we next quantified the number of neutrophils and macrophages that were in contact with fungal cells. Throughout imaging of both *A. fumigatus* and *C. albicans* infections, macrophages regularly harbored phagocytosed fungi and were observed interacting with extracellular fungi, whereas direct neutrophil-fungal interactions were less common (Fig. 3A and B). Quantification of the number of immune cells interacting with fungi per fish showed that macrophages were engaged with fungal cells around twice as often as neutrophils, possibly suggesting a more direct role for macrophages in controlling fungal infection in burn wounds, potentially via phagocytosis (Fig. 3C and D).

### Macrophage depletion leads to increased fungal burden in the burn wound

**Fig 4:**
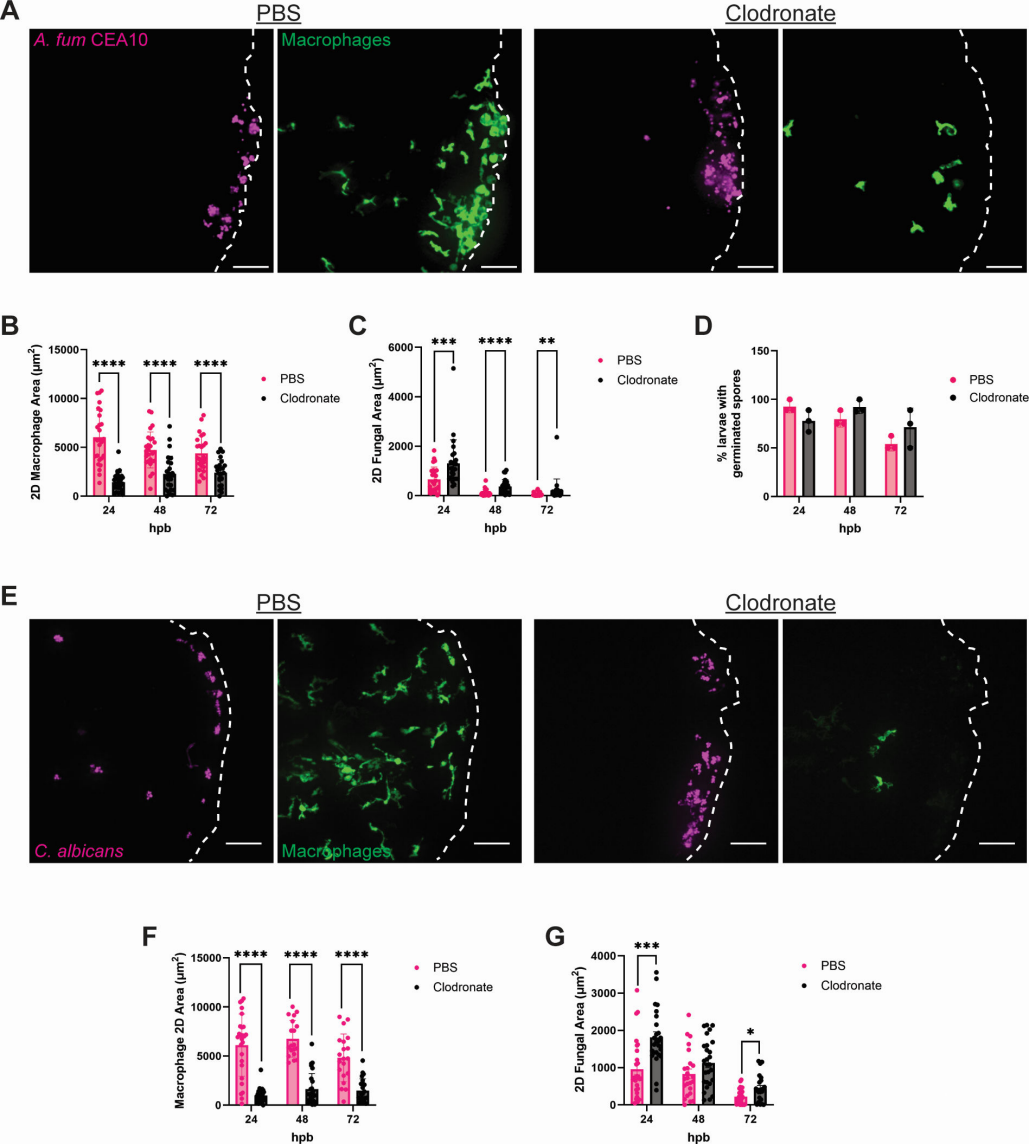
Macrophage depletion leads to increased fungal burden in the burn wound. (A) Representative images of clodronate liposome or PBS liposome treated *mpeg-GFP* larvae infected with *A. fumigatus* CEA10 at 24 hpb. Images represent maximum intensity projections of z-stacks (scale bar is 50 µm). Dashed white lines outline tail fin edge. (B) 2D-macrophage area of *A. fumigatus* infected fish from 24 to 72 hpb. (C) 2D-fungal area of *A. fumigatus* infected fish from 24 to 72 hpb. (D) Percent of larvae with germinated *A. fumigatus* spores from 24 to 72 hpb. Each data point represents the percent of fish containing germinated spores from an individual experiment. (E) Representative images of clodronate liposome or PBS liposome treated *mpeg-GFP* larvae infected with *C. albicans* (dTomato) at 24 hpb. Images represent maximum intensity projections of z-stacks (scale bar is 50 µm). Dashed white lines outline tail fin area. (F) 2D-macrophage area of *C. albicans* infected fish from 24 to 72 hpb. (G) 2D-fungal area of *C. albicans* infected fish from 24 to 72 hpb. (For all graphs, each data point represents data from an individual fish, and results represent data pooled from three independent experiments. *n* = 24–30 larvae per condition from three independent experiments. *P < 0.05, **P < 0.01, ***P < 0.001, ****P < 0001, via multiple Mann-Whitney tests.)

Our data suggest that macrophages may be the predominant leukocyte driving fungal BWI clearance (Fig. 2 and 3). However, previous work has shown that fungal phagocytosis by macrophages often does not result in fungal clearance, as both *A. fumigatus* and *C. albicans* can germinate within and escape from macrophages (25–28). To better elucidate the contribution that macrophages play in BWI resolution, we transiently depleted macrophages by injecting clodronate liposomes into the caudal vein of 2 dpf larvae expressing fluorescently labeled macrophages (*mpeg-GFP*) and proceeded to burn and infect larvae at 3 dpf (29). For *A. fumigatus* infection, we found that clodronate effectively depleted macrophages and resulted in a significant increase in fungal burden at all time points compared with PBS liposome-injected larvae (Fig. 4A through C). Interestingly, we did not observe significant differences in germination, as both germ tubes and hyphae were observed throughout the experiment (Fig. 4D). Similar to *A. fumigatus,* clodronate-injected fish infected with *C. albicans* showed a significant increase in fungal burden at 24 hpb (Fig. 4E through G), although this difference was lost by 48 hpb. Altogether, our data support an important role for macrophages in controlling fungal burden, especially during the first 24 hpb.

### Neutrophil deficiency leads to increased hyphal growth within burn tissue

**Fig 5:**
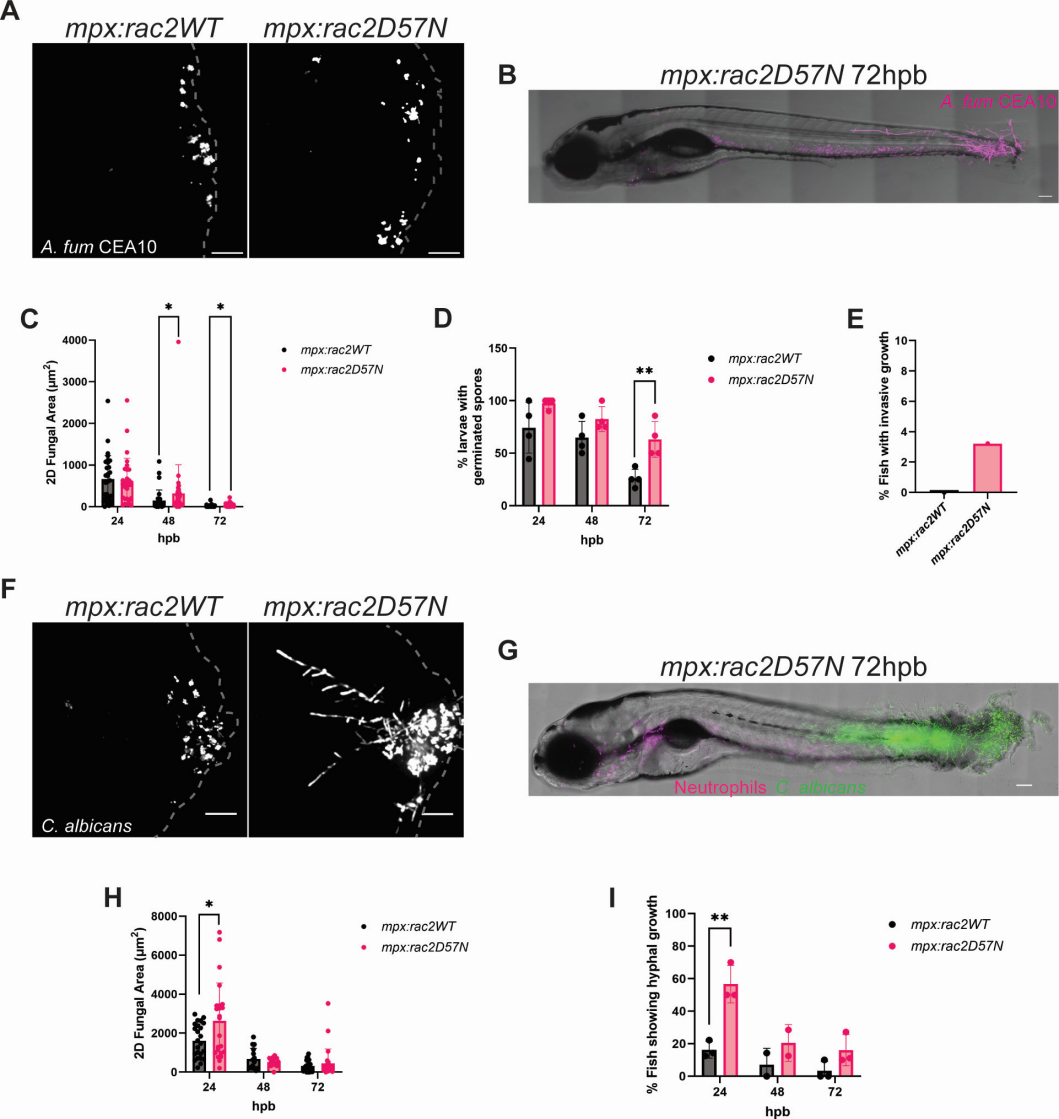
Neutrophil deficiency leads to increased hyphal growth and invasion. (A) Representative images at 24 hpb of *mpx:rac2D57N* or *mpx*:*rac2* wild-type larvae infected with *A. fumigatus* CEA10 (RFP). Images represent maximum intensity projections of z-stacks (scale bar 50 µm). Dashed gray lines outline tail fin area. (B) Representative image of entire *mpx:rac2D57N* larvae infected with *A. fumigatus* CEA10 at 72 hpb (scale bar is 100 µm). (C) 2D-fungal area of *A. fumigatus* infected fish from 24 to 72 hpb. Each data point represents data from an individual fish, and results represent data pooled from three independent experiments. (D) Percent of larvae with germinated *A. fumigatus* spores from 24 to 72 hpb. Each data point represents the percent of fish containing germinated spores from an individual experiment. (E) Percent of fish with invasive *A. fumigatus* hyphal growth extending beyond the notochord and growing toward the head at 72 hpb. Bar represents the sum of fish showing invasive growth from four experiments. (F) Representative images at 24 hpb of 3 dpf *mpx:rac2D57N* (mCherry) or *mpx*:*rac2* (mCherry) wild-type larvae infected with *C. albicans* (mNeon). Images represent maximum intensity projections of z-stacks (scale bar is 50 µm). Dashed gray lines outline tail fin edge. (G) Representative image of entire *mpx:rac2D57N* (mCherry) larvae infected with *C. albicans* (mNeon) at 72 hpb (scale bar is 100 µm). (H) 2D-fungal area of *C. albicans* infected fish from 24 to 72 hpb. Each data point represents data from an individual fish, and results represent data pooled from three independent experiments. (I) Percent of fish with *C. albicans* hyphal growth from 24 to 72 hpb. Each dot represents a percent from an individual experiment. (*n* = 24–30 larvae per condition from three independent experiments. *P < 0.05, **P < 0.01, via multiple Mann-Whitney tests or multiple Student’s *t*-tests.)

Although our data place macrophages as the primary leukocyte directly interacting with fungi at the burn-wound interface, there is ample evidence showing neutrophils as key players in the innate immune response against fungal infections (25, 26, 30–32). Indeed, neutrophil deficiency in human patients has been associated with invasive fungal growth and increased risk for burn wound infections, and *ex vivo* burn wound models using human tissue have shown an important role for neutrophils in the epidermal antifungal immune response (9, 33, 34). To assess the role of neutrophils during zebrafish fungal BWI, we infected larvae with impaired neutrophil function that express a dominant-negative Rac2 (RacD57N) protein under the regulatory control of a neutrophil-specific promoter (*mpx:rac2D57N*). These neutrophils are not able to leave the circulation and are therefore unable to migrate to burn tissue at the infection site (35). Previous work using *mpx:rac2D57N* fish demonstrated that host survival is decreased during *A. fumigatus* hindbrain infection, as well as for *C. albicans* swim-bladder infection (32, 36). Interestingly, we found increased fungal burden for *A. fumigatus* infected *mpx:rac2D57N* fish at 48 and 72 hpb compared with WT (Fig. 5A through C). A significant increase in germination and hyphal development was also observed in infected *mpx:rac2D57N* fish at 72 hpb (Fig. 5D). Surprisingly, a small number (3.3%) of *mpx:rac2D57N* fish presented invasive hyphal growth that extended from the wound toward the head by penetrating muscle tissue of the dorsal fin (Fig. 5B and E). Although rare, this is indicative of severe infection, in which *A. fumigatus* was not only able to colonize viable tissue but to also invade beyond the infection site.

In contrast to *A. fumigatus*, mutant fish infected with *C. albicans* had a significant increase in fungal burden at 24 hpb compared to WT (Fig. 5F through H). The increase in fungal burden largely correlated with extensive hyphal networks within the tail fins of *mpx:rac2D57N* infected fish by 24 hpb (Fig. 5I); ~55% of *mpx:rac2D57N* fish showed branched *C. albicans* hyphae extending throughout the tail fin by 24 hpb that were not observed in immunocompetent fish. Surprisingly, by 72 hpb, invasive hyphal growth was absent in many of these fish, suggesting a redundant role for other immune cells in controlling fungal hyphal formation. However, hyphae extending beyond the notochord and growing toward the head of the zebrafish were still seen in ~15% of the *mpx:rac2D57N*-infected fish (Fig. 5G and I), making it a more common occurrence compared with *A. fumigatus* infection (Fig. 5E). Together, these findings highlight an essential role for neutrophils in controlling invasive hyphal growth during infection with both *A. fumigatus* and *C. albicans*.

### Impaired macrophage and neutrophil function increases invasive fungal infection

**Fig 6:**
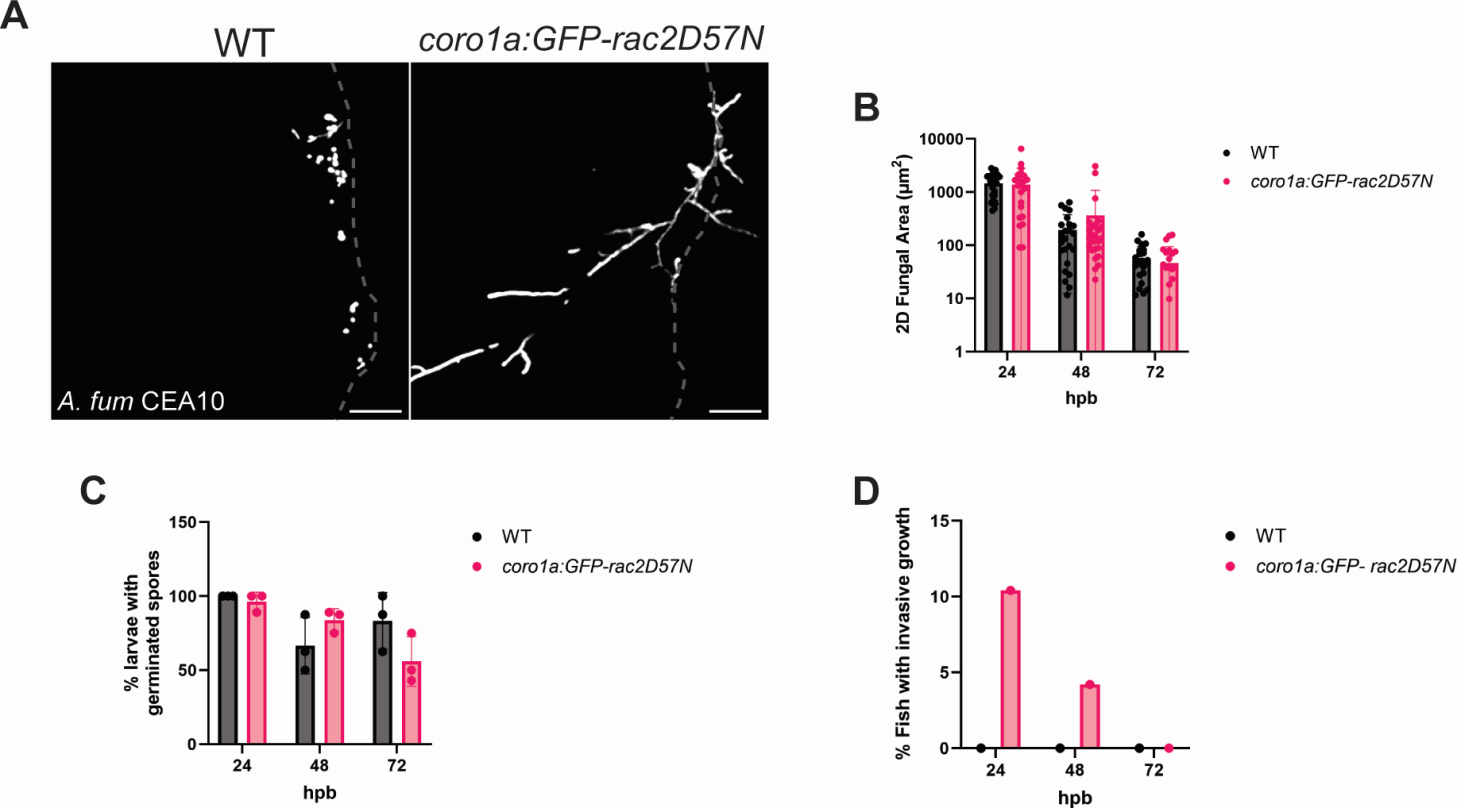
Impaired macrophage and neutrophil function results in increased *A. fumigatus* invasive hyphal growth. (A) Representative images of *coro1a:GFP-rac2D57N* and wild-type larvae infected with *A. fumigatus* CEA10 (RFP) at 24 hpb. Images represent maximum intensity projections of z-stacks (scale bar is 50 µm). Dashed gray lines outline tail fin edge. (B) 2D-fungal area of *A. fumigatus* infected fish from 24 to 72 hpb. Each data point represents data from an individual fish, and results represent data pooled from three independent experiments. (C) Percent of larvae with germinated *A. fumigatus* spores at 24–72 hpb. Each data point represents the percent of fish containing germinated spores from an individual experiment. (D) Percent of fish with invasive hyphal growth at 24–72 hpb. Bars represent the sum of fish with invasive hyphal growth from three experiments. (*n* = 24–30 larvae per condition from three independent experiments. Statistics were run via multiple Mann-Whitney tests or multiple Student’s *t*-tests.)

Our data have shown a role for both macrophages and neutrophils in controlling both *A. fumigatus* and *C. albicans* infections. Therefore, we hypothesized that infection in fish with deficiencies in both cell types would further exacerbate the disease phenotype. To test this, we used a zebrafish line that expresses a dominant-negative Rac2D57N in leukocytes (*coro1a:GFP-rac2D57N*) (37). Thus, both macrophages and neutrophils expressing this mutation have migration deficiencies that will impede their movement to the BWI site. Surprisingly, infection of *coro1a:GFP-rac2D57N* fish with *A. fumigatus* did not appear to result in increased fungal burden or germination rates (Fig. 6A through C) but did result in an increase in the number of fish showing invasive hyphal growth extending beyond the notochord as early as 24 hpb. Indeed, 10% and 5% of all fish imaged showed hyphal growth extending past the notochord and moving toward the head at 24 and 48 hpb, respectively (Fig. 6D). Therefore, loss of both macrophages and neutrophils further impacts invasive hyphal growth of *A. fumigatus*, although it appears to be modest.

**Fig 7:**
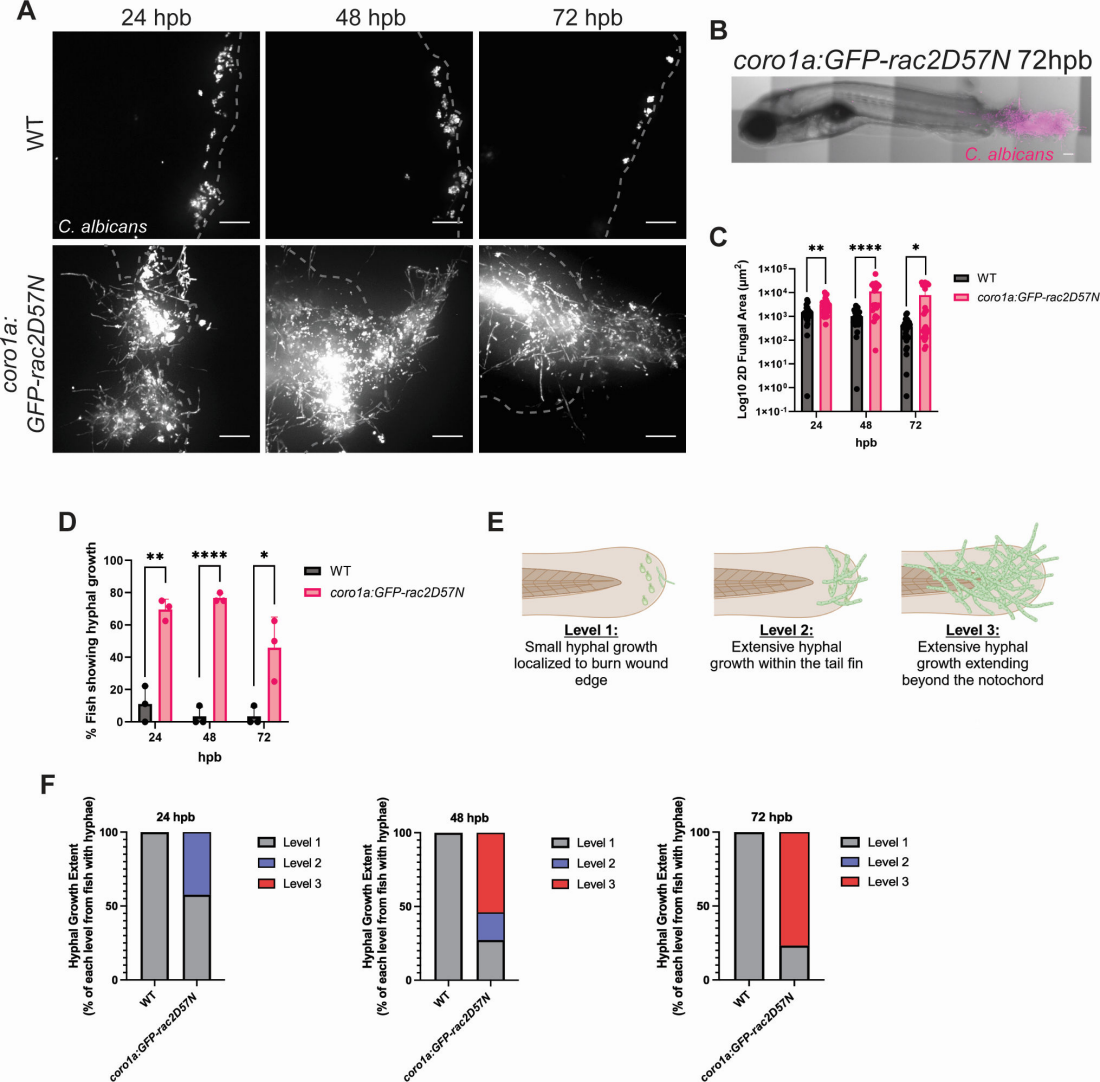
Impaired macrophage and neutrophil function results in uncontrolled growth and invasion of *C. albicans* in thermal injury. (A) Representative images of *coro1a:GFP-rac2D57N* or wild-type larvae infected with *C. albicans* (dTomato) and imaged at 24, 48, and 72 hpb. Images represent maximum intensity projections of z-stacks (scale bar is 50 µm). Dashed gray lines outline tail fin edge. (B) Representative image of entire *coro1a:GFP-rac2D57N* larvae infected with *C. albicans* (dTomato) at 72 hpb (scale bar is 100 µm). (C) 2D-fungal area of *C. albicans* infected fish from 24-72 hpb. Each data point represents data from an individual fish, and results represent data pooled from three independent experiments. (D) Percent of fish showing *C. albicans* hyphal growth from 24 to 72 hpb. Each data point represents the percent of fish containing hyphae from an individual experiment. (D) Schematic of hyphal growth classification. Level 1 indicates small and controlled hyphal growth present at the burn wound edge. Level 2 is denoted as extensive hyphal growth within the tail fin. Level 3 is classified as extensive and invasive hyphal growth that penetrates beyond the notochord and extends toward the head of the fish. (F) Percent of fish showing hyphal growth within each classification level (1–3). Bar colors represent the different levels and are derived from the mean of 3 individual experiments. (*n* = 24–30 larvae per condition from three independent experiments. *P < 0.05, **P < 0.01, ****P < 0001, via multiple Mann-Whitney tests or multiple Student’s *t*-tests.)

Unlike *A. fumigatus*, infection in *coro1a:GFP-rac2D57N* fish with *C. albicans* showed a significant increase in fungal burden at all the times analyzed (Fig. 7A through C). This was accompanied by robust hyphal development as early as 24 hpb that persisted throughout infection. Overall, between 50% and 75% of *C. albicans*-infected *coro1a:GFP-rac2D57N* zebrafish showed hyphal growth of varying degrees throughout the infection (Fig. 7D). We then further categorized hyphal presence based on the severity of invasive growth (Fig. 7E). At 24 hpb, the hyphal growth was either small hyphae localized to the burn wound edge, denoted as level 1, or extensive and branched hyphal growth that was localized within the tail fin, denoted as level 2 (Fig. 7F). However, as the infection progressed, the percentage of fish displaying hyphae extending past the notochord and toward the head of the fish, denoted as very invasive level three hyphal growth, increased. Indeed, ~55% and ~80% of hyphal growth at 48 hpb and 72 hpb, respectively, was classified as severely invasive (Fig. 7F). Therefore, it appears that both macrophages and neutrophils are essential to control *C. albicans* during infection. However, it is important to note that *coro1a:GFP-rac2D57N* fish also showed an innate sensitivity to burn wound injury, and excessive tissue necrosis and death were observed even in the absence of infection (Fig. S2). Fungal presence did not appear to further increase death, and it seems likely that the excessive tissue damage observed in *coro1a:GFP-rac2D57N* fish allowed *C. albicans* to more effectively colonize and invade the burn wound niche.

### Increased β(1,3)-glucan exposure attenuates *C. albicans* burn wound colonization in zebrafish

**Fig 8:**
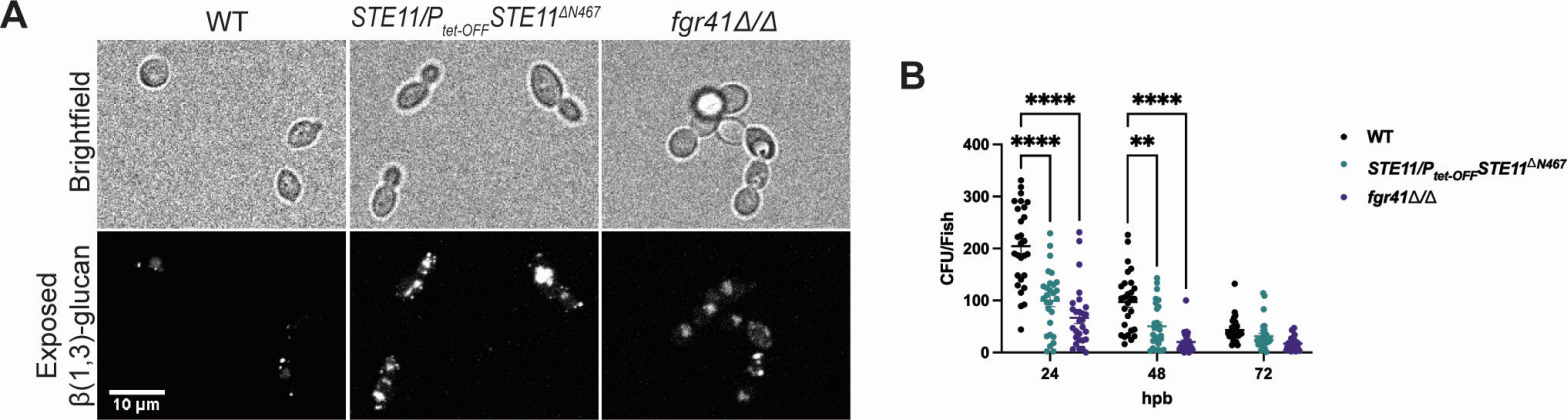
Increased β-glucan exposure attenuates burn wound colonization in zebrafish. (A) Representative microscopy images of β(1,3)-glucan exposure of wild-type, *STE11/P_tet-OFF_STE11*^Δ^*^N467^* and *fgr41*Δ/Δ *C. albicans* strains. The scale bar indicates 10 µm. (B) CFUs per fish from wild-type zebrafish larvae that were infected with either *C. albicans* wild type or mutants that show increased β(1,3)-glucan exposure (*STE11/P_tet-OFF_STE11*^Δ^*^N467^* and *fgr41*Δ/Δ). Each data point represents data from an individual fish. Results represent data pooled from three independent experiments. *n*= 24-30 larvae per condition. (**P < 0.01, ****P < 0001, by a two-way ANOVA with Šidák’s multiple comparisons test.)

Both macrophages and neutrophils are needed to control burn wound infection by *C. albicans* (Fig. 7), and we hypothesized that infection with fungal mutants that have an attenuated ability to evade host immune system recognition would result in enhanced fungal clearance within this niche. *C. albicans* mutants with a hyperactive Cek1 mitogen-activated protein kinase pathway (*STE11/P_tet-off_STE11*^Δ^*^N467^*) or that are deficient in the cell wall protein Fgr41 (*fgr41*Δ/Δ) display increased exposure of the immunogenic cell wall epitope β(1,3)-glucan (Fig. 8A), induce increased TNFα production by macrophages *in vitro* and display virulence attenuations that are host immune system dependent during systemic infection in mice (38–40). Therefore, we predicted that these mutants would also be attenuated in their ability to colonize burn tissue within an immunocompetent zebrafish burn model. Indeed, WT fish infected with either *STE11/P_tet-off_STE11*^Δ^*^N467^* or *fgr41*Δ/Δ showed significantly less fungal burden than WT *C. albicans* infected fish at both 24 hpb and 48 hpb (Fig. 8B). Thus, masking immunogenic β(1,3)-glucan is an important mechanism utilized by *C. albicans* to successfully colonize burn tissue.

## DISCUSSION

Fungal burn wound infections are an understudied but significant complication that increases the risk of disease and mortality in patients. The challenge of finding suitable animal models to study the biology behind fungal infections during burn injury represents a major obstacle to better understanding disease kinetics. Various animal models exist to study burn infections, but both species-specific differences and economic constraints complicate their use. For example, mice have a looser skin architecture with a denser hair structure than humans and primarily heal from thermal injury via contraction, whereas humans undergo re-epithelization (13). Porcine skin architecture is anatomically closer to humans and heals from thermal wounds in a similar manner (13). However, this model is expensive and therefore cost prohibitive for general use. Zebrafish are a genetically tractable and cost-effective alternative to the existing animal models to study BWIs. Zebrafish larval skin structure is comprised of stratified epithelium with two layers of keratinocytes and a dermis and responds to thermal injury by mild contraction followed by re-epithelization (41, 42). However, unlike mammalian models, the optical transparency of zebrafish larvae allows for intravital imaging that facilitates the study of tissue repair, regeneration, and host-pathogen interactions in viable tissue throughout the infection process (19, 20, 22, 41). By exploiting these characteristics, we developed a fungal burn wound infection model using zebrafish larvae to better elucidate host-fungal interaction dynamics within the burn wound niche.

By combining live imaging and quantification of fungal burden, we demonstrate that both *A. fumigatus* and *C. albicans* successfully colonize and are cleared from immunocompetent zebrafish larvae (Fig. 1), therefore providing an efficient model for acute wound infection. Both macrophages and neutrophils are recruited to the site of trauma (Fig. 2). Fungal colonization results in increased neutrophil recruitment in response to both pathogens, but only *C. albicans* induces increased macrophage infiltration as well. Thus, it appears that each fungal isolate has distinct effects on the host immune response following burn injury. It is possible that the divergent metabolic nature between the pathogens may account for these differences. *C. albicans* yeast cells are active and appear to readily undergo morphological transitioning to hyphae at the early stages (2–6 hpb) of burn wound colonization (Fig. 1). Hyphae are both more invasive than yeast cells and have increased exposure of β-glucan moieties within their cell wall that may further exacerbate the host immune response (43–45). By contrast, *A. fumigatus* conidia are generally considered metabolically dormant, and germination does not occur until 24–48 hpb (Fig. 1) (46). Structurally, the spore surface is covered by hydrophobins that mask underlying immunogenic cell wall epitopes and aids in immune cell evasion (47, 48). Therefore, the delayed germination rate of *A. fumigatus* spores likely contribute to reduced immune cell infiltration compared with *C. albicans*.

**Fig 9:**
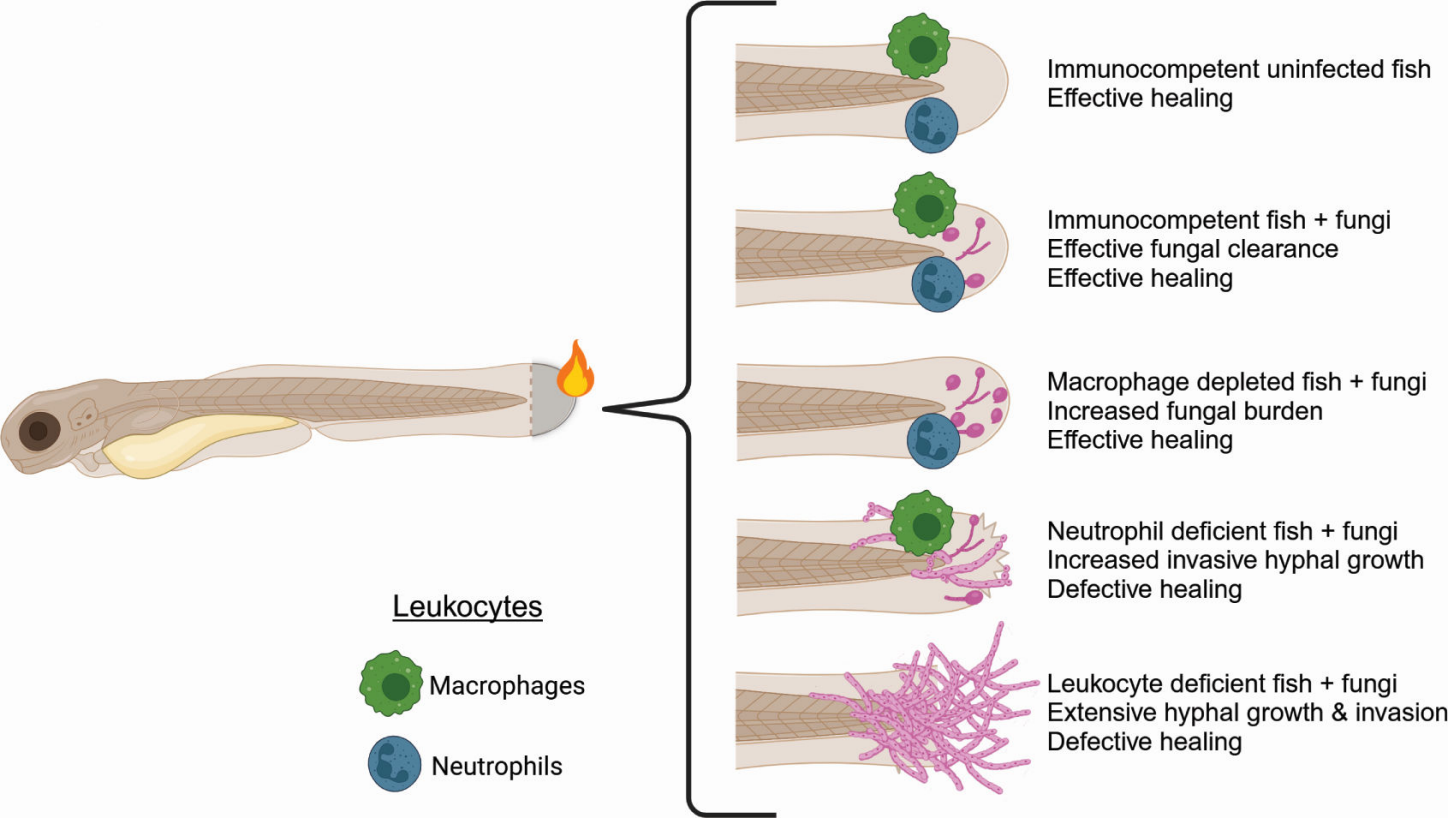
Model of fungal burn wound infection in zebrafish.

Our findings suggest distinct roles for neutrophils and macrophages at different stages of infection (Fig. 9). Depletion of macrophages results in increased fungal burden at early stages of infection (Fig. 4), whereas infection in neutrophil-defective zebrafish results in invasive hyphal growth by both fungi (Fig. 5). However, both fungal burden and hyphal presence during *C. albicans* infections are largely restored to WT levels at the later stages of infection in both macrophage- or neutrophil-deficient zebrafish, suggesting redundant functions. Infection in *coro1a:GFP-rac2D57N* zebrafish larvae, which have defective neutrophils and macrophages, increase both fungal burden and invasive hyphal growth with *C. albicans* (Fig. 7), supporting the idea that macrophages and neutrophils have synergistic and partially redundant roles in antifungal immunity in burns.

The observation that infection in fish with defective neutrophils results in increased invasive hyphal development (Fig. 5) recapitulates findings from both clinical reports and *ex vivo* human skin infection models (9, 34). However, our findings that the loss of macrophages impacts fungal colonization (Fig. 4) raise interesting questions about the role of macrophages in fungal control during thermal injury. *C. albicans* yeast cells can remain viable, divide, and be transported within macrophages to distant sites from the initial infection (49). Studies with *A. fumigatus* using the zebrafish hindbrain infection model have similarly shown that macrophages phagocytose but often do not kill conidia and actually delay clearance by inhibiting germination and subsequent hyphal killing by neutrophils (26). However, it was recently reported that macrophage-specific Rac2 rescues control of invasive hyphal growth by *A. fumigatus* in pan-Rac2 deficient fish during hindbrain infection (50). Our data further indicate that macrophages can control hyphal growth in the absence of neutrophils, demonstrating that macrophages do play an important role in antifungal immunity. A caveat is that there may be off-target effects of clodronate treatment that contribute to some of the observed phenotypes, since studies in mice suggest that clodronate liposomes may also have effects on neutrophil function (51).

Our findings highlight a distinction between colonization and infection. In humans, skin infection is characterized by fungal penetration to variable depths within viable tissue, along with angioinvasion (52–54). Colonization is limited to the wound surface and proliferation at the interface between viable and non-viable tissue (10, 52, 55). In immunocompetent and macrophage-depleted fish, fungi were highly localized and displayed minimal hyphal development, which suggests colonization of the tissue. However, neutrophil deficiency was associated with fungal invasion of neighboring tissues. Although zebrafish larvae do not fully develop vasculature until later stages of development, we propose that the invasive hyphal growth observed in larvae with impaired neutrophil function represents infected tissue and supports a key role of neutrophils in preventing invasive growth within burns (56). However, it is important to note that infections in this model occur at 28.5°C, which is the optimal incubation temperature for zebrafish. The external surface of human skin ranges from 30.1°C to 34.7°C (57), and fungi more readily transition to hyphae at temperatures closer to 37°C. Despite the lower temperature, fungi still effectively germinated and developed hyphae that penetrated neighboring tissue, but it is possible that higher infection temperatures may also alter the kinetics of these interactions.

To test the utility of this BWI model for identifying fungal mechanisms of tissue colonization, we infected fish with *C. albicans* mutants that had increased β(1,3)-glucan exposure within their cell walls (Fig. 8). β(1,3)-Glucan is highly immunogenic, and these mutants have been previously shown to induce robust pro-inflammatory cytokine production by macrophages in a largely dectin-1 dependent manner *in vitro* and show immune system-dependent virulence attenuations during systemic infections in mice (38–40). Our results show limited tail fin colonization with unmasked *C. albicans* mutants (Fig. 8), similar to the phenotypes observed in mice. These findings suggest that masking of β(1,3)-glucan is necessary for persistent tissue colonization in larval burn wounds and highlights the efficacy of this model for studying fungal mechanisms of tissue colonization during burn injury. However, both mutants also display increased levels of chitin in their cell wall (39, 40). Although evidence suggests that chitin has an immunosuppressive impact on host immune signaling, it is possible that changes to this cell wall epitope may also have an impact on the observed phenotypes (58). Therefore, further analysis should be performed to better characterize the host response to infection with other cell wall mutants within the context of BWIs, with the potential for these findings to have broader implications across different infection models and disease manifestations.

## MATERIALS AND METHODS

### Zebrafish husbandry and maintenance

Adult zebrafish were maintained under a light/dark cycle of 14/10 h, as previously described (36). For experiments, embryos were collected, screened, and maintained at 28.5°C. All zebrafish lines utilized in this study are listed in Table S1.

### Fungal strains growth and conditions

All fungal strains used in this study are listed in Table S2. *A. fumigatus* and *C. albicans* strains were maintained as glycerol stocks at −80°C. For *A. fumigatus,* the strain was activated before every experiment by streaking on a plate of glucose minimal media (GMM) and incubating for 3 days in darkness at 37°C (59). Conidia were harvested in 0.01% Tween-water by scraping with an L-shaped spreader and immediately filtered through sterile Miracloth. To prepare for burn wound infection, conidia solution was centrifuged and washed three times in a sterile 1**×** PBS solution. After counting conidia, the solution was resuspended in PBS. The appropriate volume to achieve 2 × 10^7^ spores/mL was then added to a conical tube with 5 mL of E3 without methylene blue (E3-MB) for later use.

For *C. albicans,* the strains were activated on YPD agar plates (1% yeast extract, 2% peptone, 2% dextrose, 2% agar) from glycerol stocks. One day prior to infection, overnight cultures in liquid YPD media were started and left to incubate for ~16 h while shaking at 225 rpm at 30°C. Following incubation, cultures were transferred to a 15 mL conical tube and centrifuged at 3,500 rpm for 5 min. Following centrifugation, the supernatant was aspirated, and the fungal cell pellet was washed 2 times with 10 mL of 1**×** PBS. After washing, the fungal cell pellet was resuspended in 5 mL of E3-MB media, counted on a hemocytometer, and diluted to a concentration of 2 × 10^7^ cells/mL in 6 mL of E3-MB media.

### Thermal injury and infection

Infections were done using 3 dpf larvae for all experiments. Before every experiment, 3 dpf larvae were anesthetized in E3-MB media containing 0.2 mg/mL Tricaine (ethyl 3-aminobenzoate, Sigma). For thermal injury and infection, larvae were placed in 10 mL of E3-MB + Tricaine in a 60 mm tissue culture-treated dish (Corning) (8–10 larvae/dish). A fine-tip cautery pen (Bovie, Symmetry Surgical) was used to burn the caudal fin without injuring the notochord (19). Immediately after burn, 5 mL of liquid was removed from the dish and replaced with either 5 mL of the fungal solution (containing 2 × 10^7^ cells/mL of fungi) previously prepared to achieve a final concentration of 1 × 10^7^ cells/mL or 5 mL of fresh E3-MB as a control. Larvae were then slowly rocked and kept in solution for 1 hour. Afterward, larvae were rinsed five times with E3-MB and incubated at 28.5°C for further analysis.

### Clodronate liposome injection

Transgenic larvae with fluorescent macrophages (*mpeg:GFP*) were microinjected at 2 dpf into the caudal vein plexus with 2 nL of clodronate or PBS liposomes (Liposoma) with 0.1% phenol red for visualization, as previously described (29). Infection then proceeded at 3 dpf as described above.

### Fungal burden enumeration by colony-forming units

For *A. fumigatus* and *C. albicans*, individual larvae were collected at 24, 48, and 72 hpb and placed in 1.5 mL microcentrifuge tubes with 90 ul of 1**×** PBS with 50 μg/mL gentamycin and 50 μg/mL kanamycin. Larvae were placed on ice for 30 minutes and then homogenized for 15 seconds in a mini-beadbeater-16 (BioSpec) at maximum speed. The entire volume was plated on solid GMM (*A. fumigatus*) or YPD (*C. albicans*) plates, incubated at 30**–**37 °C for 2 days, and then, CFUs were counted. Three biological replicates were run for all cell lines tested, and the total number of fish used is listed in the appropriate figure legends. Statistical analysis was performed using either a one-way ANOVA with Tukey’s multiple comparison analysis or a two-way ANOVA with Šidák’s multiple comparisons test (GraphPad Prism, v7.0c software).

### Live imaging acquisition

For live imaging acquisition, larvae were anesthetized and embedded flat on their side in a 35 mm glass bottom dish (CellVis) by adding 1%**–**2% low melting point agarose on top. Z-series images (4 µm slices) of the tail fin were acquired on a spinning disk confocal microscope (CSU-X; Yokogawa) with a confocal scan head on a Zeiss Observer Z.1 inverted microscope with a Photometrics Evolve EMCCD camera. ZEN 2.6 software was used for acquisition. Tiles and Z-series were acquired and stitched using ZEN software for whole larvae images.

### Imaging analyses and processing

Images were processed and analyzed using FIJI ImageJ (60). Maximum intensity projections of Z-series images were used for representative images and for further analysis. Fungal burden and leukocyte recruitment were analyzed by manual thresholding and measuring 2D area of the corresponding fluorescent signal. Germination for *A. fumigatus* was scored as the presence or absence of germinated conidia (germ tube or hyphae). Invasive hyphal growth was quantified by the presence of branched hyphal networks extending beyond the tail fin edge toward the caudal vein of the fish. For immune cells contacting fungi quantification, each image in the whole Z-series was analyzed, and the number of macrophages or neutrophils physically contacting fungal cells (intra- and extra-cellularly) within the same image plane was counted. Counts were then pooled for all planes to generate contact numbers per fish. In all representative images, brightness and contrast were adjusted for visual purposes only, and no alterations were made to images prior to analysis. For measuring tissue regrowth, the total fin tissue area distal to the notochord was outlined using the polygon tool (19). Three biological replicates were run for all experiments, and the total number of fish used is listed in the appropriate figure legends. Once data were collected, distribution normality was assessed with the use of a D’Agostino-Pearson omnibus normality test, and statistical significance was determined via either multiple Student’s *t*-tests or multiple Mann–Whitney tests (GraphPad Prism, v7.0c software)

### β(1,3)-Glucan staining

Fungal β(1,3)-glucan was stained as previously described, with modification to the secondary antibody used in this study (61). Here, a secondary rat anti-mouse fluorescein isothiocyanate (FITC)-conjugated antibody (Biolegend; 406001) was used at a 1:250 dilution for staining. Following staining, the cells were imaged on a Zeiss Observer Z.1 inverted microscope, and images were processed via Fiji ImageJ.

## SUPPLEMENTAL MATERIAL

Supplemental Figures mbio.03480-24-s0001.pdf Figures S1 and S2.

Table S1 mbio.03480-24-s0002.docx Fish lines used in this study.

Table S2 mbio.03480-24-s0003.docx Fungal strains used in this study.

ASM does not own the copyrights to Supplemental Material that may be linked to, or accessed through, an article. The authors have granted ASM a non-exclusive, world-wide license to publish the Supplemental Material files. Please contact the corresponding author directly for reuse.
