# Supplementary material for: Larval zebrafish burn wound infection model reveals conserved innate immune responses against diverse pathogenic fungi
Source: mBio. 2025 Apr 8;16(5):e03480-24. doi: 10.1128/mbio.03480-24 (PMC12077223; doi:10.1128/mbio.03480-24)
Supplement: Supplemental Figures — Figures S1 and S2. [file mbio.03480-24-s0001.pdf]

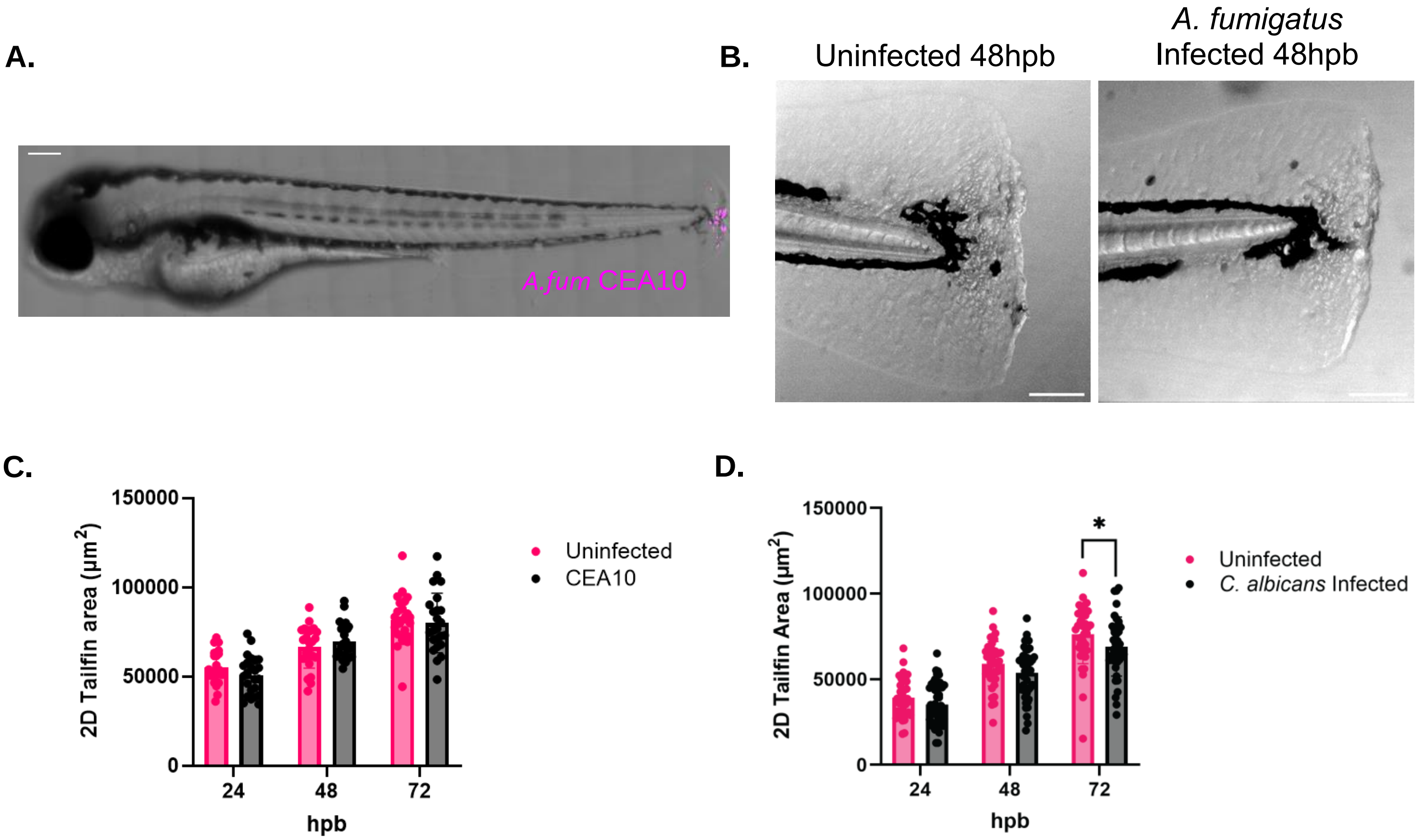
A.
A.fum CEA10
B.
Uninfected 48hpb
A. fumigatus
Infected 48hpb
C.
2D Tailfin area (µm²)
150000
100000
50000
0
24
48
72
hpb
Uninfected
CEA10
D.
2D Tailfin Area (µm²)
150000
100000
50000
0
24
48
72
hpb
*
Uninfected
C. albicans Infected

Supplemental Fig 1. **Tail fin regrowth is not impaired by infection.**

3-dpf wild-type larvae were injured and infected with either *A. fumigatus* CEA10 (RFP) or *C. albicans* (dTomato). Control fish were uninfected. (A) Representative image of entire wild-type larvae infected with *A. fumigatus* CEA10 (RFP) (scale bar 25 µm). Note fungi localized only at the edge of the tail fin (B) Representative brightfield images of larvae infected with *A. fumigatus* CEA10 or uninfected at 48hpb. (C) 2D-tail fin area at 24-72 hpb of larvae uninfected or infected with *A. fumigatus* CEA10. (D) 2D-tail fin area at 24-72 hpb of larvae uninfected or infected with *C. albicans*. (For all graphs, each data point represents data from an individual fish. Results represent data pooled from 3 independent experiments. n= 24-30 larvae per condition. Statistics determined by multiple Mann-Whitney tests or multiple student's t-tests.)

A.

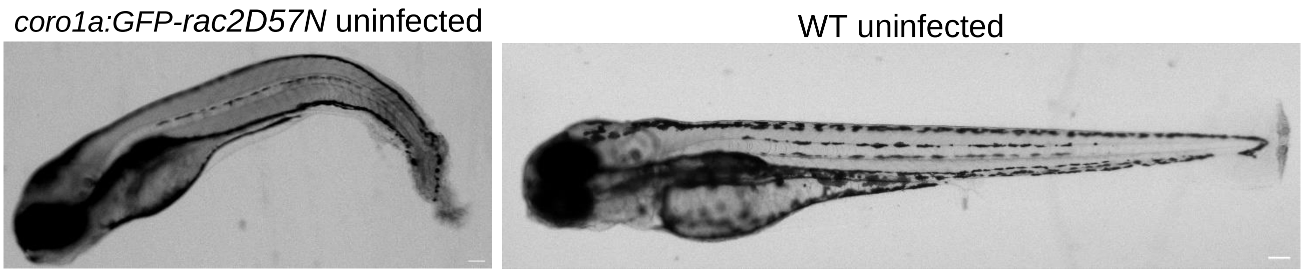
coro1a:GFP-rac2D57N uninfected
WT uninfected

B.

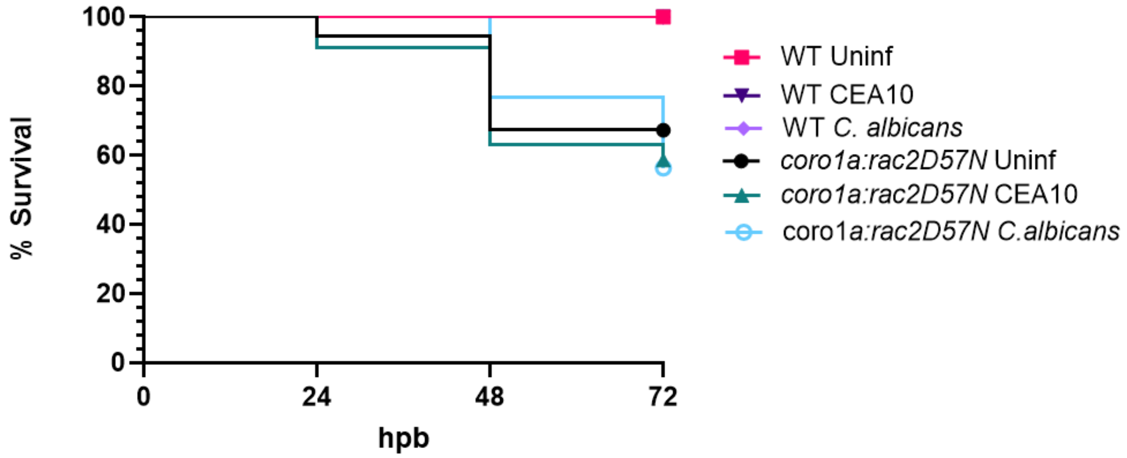
100
80
60
40
20
0
% Survival
0
24
48
72
hpb
WT Uninf
WT CEA10
WT *C. albicans*
coro1a:rac2D57N Uninf
coro1a:rac2D57N CEA10
coro1*a:rac2D57N C.albicans*

Supplemental Fig 2. Macrophage and neutrophil deficient larvae are more susceptible to burn wound.

3-dpf *coro1a:GFP-rac2D57N* (GFP) or wild-type larvae were thermally injured and infected with *A. fumigatus* CEA10 or *C. albicans* and imaged at 24 hpb. (A) Representative brightfield image of entire uninfected wild-type or *coro1a*:Rac2D57N infected larvae (scale bar 200µm). (B) Kaplan-Meier survival curve of infected versus uninfected wild-type and *coro1a:GFP-rac2D57N* larvae.
