# Supplementary material for: Larval zebrafish burn wound infection model reveals conserved innate immune responses against diverse pathogenic fungi
Source: mBio. 2025 Apr 8;16(5):e03480-24. doi: 10.1128/mbio.03480-24 (PMC12077223; doi:10.1128/mbio.03480-24)
Supplement: Table S1 — Fish lines used in this study. [file mbio.03480-24-s0002.docx]

| **Line** | **Description** | **Reference** |
| --- | --- | --- |
| WT (AB) | Wild-type line | ZIRC |
| Tg(*lyz*:*BFP*/*mpeg1*:*GFP*) | Wild type with BFP-expressing neutrophils and GFP-expressing macrophages | (1) |
| Tg(*mpeg1*:*GFP*) | Wild type with GFP-expressing macrophages | ZIRC |
| Tg(*lyz*:*GFP*) | Wild type with GFP-expressing neutrophils | ZIRC |
| Tg(*mpx*:*mCherry*-2A-rac2D57N) | Rac2D57N mutation and mCherry-expressing neutrophils | (2) |
| Tg(*mpx*:*mCherry*-2A-rac2WT) | Wild-type Rac2 and mCherry-expressing neutrophils | (2) |
| Tg(*coro1a:GFP*-rac2D57N)^psi92Tg^ | Rac2D57N mutation and GFP expressing macrophages and neutrophils | (3) |

**Table S1: Fish lines used in this study**

**References**

(1) Rosowski EE, Raffa N, Knox BP, Golenberg N, Keller NP, Huttenlocher A. 2018. Macrophages inhibit Aspergillus fumigatus germination and neutrophil-mediated fungal killing. PLoS Pathog 14:e1007229.

(2) Deng Q, Yoo SK, Cavnar PJ, Green JM, Huttenlocher A. 2011. Dual roles for Rac2 in neutrophil motility and active retention in zebrafish hematopoietic tissue. Dev Cell 21:735–745.

(3) Ramakrishnan G, Miskolci V, Hunter M, Giese MA, Münch D, Hou Y, Eliceiri KW, Lasarev MR, White RM, Huttenlocher A. 2024. Real-time imaging reveals a role for macrophage protrusive motility in melanoma invasion. Journal of Cell Biology 224:e202403096.
