# Supplementary material for: Larval zebrafish burn wound infection model reveals conserved innate immune responses against diverse pathogenic fungi
Source: mBio. 2025 Apr 8;16(5):e03480-24. doi: 10.1128/mbio.03480-24 (PMC12077223; doi:10.1128/mbio.03480-24)
Supplement: Table S2 — Fungal strains used in this study. [file mbio.03480-24-s0003.docx]

**Table S2: Fungal strains used in this study**

| **Strain** | **Parent** | **Genotype** | **Reference** |
| --- | --- | --- | --- |
| *Aspergillus fumigatus* | | | |
| TDGC1.2 | TCDN6.7 | *ΔakuB; argB-; gpdA::RFP::argB; pyrG-; fumipyrG* | (1) |
| *Candida albicans* | | | |
| Caf2-dTomato | Caf2 | *Δura3::imm434/ URA3 pENO1-dTomato-NATR* | (2) |
| SC5314-mNeon | SC5314 | *P_ENO1_-NEON-NAT* (Wild Type) | (3) |
| AWY006 | SC5314 | *LEU2/leu2∆* (Wild Type) | (4) |
| AWY080 | AWY006 | *STE11/P_tet-OFF_-STE11^∆N467^ LEU2/leu2∆* | (4) |
| SLY005 | AWY006 | *fgr41∆/∆ LEU2/leu2∆* | (5) |

**References**

(1) Schoen TJ, Calise DG, Bok JW, Giese MA, Nwagwu CD, Zarnowski R, Andes D, Huttenlocher A, Keller NP. 2023. Aspergillus fumigatus transcription factor ZfpA regulates hyphal development and alters susceptibility to antifungals and neutrophil killing during infection. PLOS Pathogens 19:e1011152.

(2) Gratacap RL, Rawls JF, Wheeler RT. 2013. Mucosal candidiasis elicits NF-κB activation, proinflammatory gene expression and localized neutrophilia in zebrafish. Disease Models & Mechanisms 6:1260.

(3) Wu Y, Du S, Johnson JL, Tung H-Y, Landers CT, Liu Y, Seman BG, Wheeler RT, Costa-Mattioli M, Kheradmand F, Zheng H, Corry DB. 2019. Microglia and amyloid precursor protein coordinate control of transient Candida cerebritis with memory deficits. Nature Communications 10:58.

(4) Wagner AS, Hancock TJ, Lumsdaine SW, Kauffman SJ, Mangrum MM, Phillips EK, Sparer TE, Reynolds TB. 2021. Activation of Cph1 causes ß(1,3)-glucan unmasking in Candida albicans and attenuates virulence in mice in a neutrophil-dependent manner. PLOS Pathogens 17:e1009839.

(5) Wagner AS, Lumsdaine SW, Mangrum MM, King AE, Hancock TJ, Sparer TE, Reynolds TB. 2022. Cek1 regulates ß(1,3)-glucan exposure through calcineurin effectors in Candida albicans. PLOS Genetics 18:e1010405.
